# Interpreter use in sustained nurse home visiting: interpreter experience and support

**DOI:** 10.1186/s12913-023-09117-z

**Published:** 2023-02-10

**Authors:** Mehrnoush Bonakdar Tehrani, Kelly Baird, Suza Trajkovski, Catherine Kaplun, Tracey Bruce, Lynn Kemp

**Affiliations:** 1 Translational Research and Social Innovation (TReSI), Level 3, 1 Campbell Street, Liverpool, NSW 2170 Australia; 2 School of Nursing and Midwifery, Western Sydney University, Sydney, Australia; 3 Ingham Institute for Applied Medical Research, 1 Campbell Street, Liverpool, NSW 2170 Australia; 4 Transforming early Education And Child Health (TeEACH) Research Centre, Westmead Innovation Quarter, Westmead, NSW 2145 Australia

**Keywords:** Child and family health nursing, Interpreters, Limited English proficiency, Migrant mothers, Sustained nurse home visiting

## Abstract

**Background:**

The aim of this study was to explore the experiences of healthcare interpreters working with child and family health nurses (CFHNs) in providing child and family health nursing (CFHN) services and sustained nurse home visiting (SNHV) programs to culturally and linguistically diverse (CALD) families with limited English proficiency.

**Methods:**

A mixed methods longitudinal research design was conducted to develop, implement and evaluate a training and practice support model for healthcare interpreters working with nurses and CALD families in providing CFHN services and SNHV programs in three major local health services in Sydney, Australia. One pre-training survey with 24 healthcare interpreters was conducted; field notes were recorded during training and implementation; and a post-implementation focus group with six healthcare interpreters was conducted. Quantitative survey data were analysed descriptively using Alchemer. The focus group was audio-recorded for transcription purposes, and this and the field notes were thematically analysed applying a socioecological framework.

**Results:**

Three themes were identified from the initial, pre-training survey: facilitate communication and delivery accurately; a bridge linking the clients and the healthcare practitioners; and make everybody feel comfortable. Practice support implementation was negatively impact by system and COVID-19 related barriers. Four themes were developed from evaluative phase of the study including: system-related issues; interpreters’ challenges; working with nurses; and client session related issues.

**Conclusion:**

Quality interpreting was favourably influenced by adequate time for interpreting the session including a pre- and post-briefing session with CFHNs, an appropriate mode of interpretation, allocation of female interpreters and the same interpreters with CALD mothers and clarity about interpreter role and cultural comfort. These strategies support the quality of communication and relationships in delivery of CFHN services and SNHV programs to CALD mothers with limited English proficiency.

## Background

Worldwide, different waves of migration have resulted in communication gaps between culturally and linguistically diverse (CALD) families and healthcare providers in host countries [1–3]. According to the Australian Bureau of Statistics, migrants comprised up to 30% of the Australian population, more than one-fifth (21%) of Australians spoke a language other than English at home, and nearly 11% identified as having limited English proficiency (limited English proficiency i.e., do not speak English well or at all) [4].

Research has consistently shown that language barriers have been identified as a major hindrance and potential cause of health inequalities for CALD families with limited English proficiency in accessing and engaging with primary healthcare [5–7]. Language incompatibilities lead to challenges for CALD families with limited English proficiency, such as higher risk of misdiagnosis; lower clients’ satisfaction; low quality of care and poorer health outcomes when compared with health care clients who speak the dominant language [8, 9]. Furthermore, excessive costs for healthcare providers in providing services for CALD families were associated with insufficient and unsatisfactory language support including lower engagement and use of primary healthcare [10].

Previous research demonstrates that using professional interpreters in healthcare settings can mitigate communication barriers, improve clients-healthcare professionals interactions, and positively influence healthcare access and quality for clients with limited English proficiency, overall lessening healthcare costs [3, 9, 11–13]. A qualitative systematic review examined working with interpreters in healthcare settings identifying that interpreters’ role differences led to sources of both tension and relational challenges [14]. For example, trust and control issues found within the relation between clients, interpreters and healthcare professionals, compromised quality of care for clients with limited English proficiency. However, much of the research to date has focused on interpreter perspectives on their roles and experiences in medical services, with limited (but growing) literature looking at the perspectives of professional interpreters working with long-term relationship-based primary care nursing services for CALD communities.

Barnes et al. [15] recognised that the use of interpreters in sustained nurse home visiting (SNHV) could assist in the development of trusting relationships between nurses and clients with limited English proficiency. A quantitative study in Sweden found that migrant women preferred using a professional interpreter because of their training, ensuring high-quality language skills and interpreter agency employment [16]. However, studies have demonstrated that interpreting services are often inadequate and inappropriate in primary health care services. For example, Mengesha et al. [17] reported that CALD women using sexual and reproductive health services were less likely to engage when male interpreters were provided due to confidentiality issues. Likewise, use of professional interpreters in maternity care was limited and compromised for Afghan migrant women with limited English proficiency, with women mostly dependant on their family members (e.g., husbands) for interpreting the session [18].

In Australia, child and family health nursing (CFHN) services and SNHV programs are designed and implemented to promote positive outcomes for mothers with young children aged up to 5 years experiencing complex life challenges [19, 20]. Child and family nurses (CFHNs) work with healthcare-provided in-person and telephone interpreter services. Previous research [19] identified that although CFHNs described some positive experiences, there were several practical and quality issues in working with interpreters. These issues include unavailability and inappropriate allocation of interpreters for interpreting services e.g., some CALD mothers’ preference for female interpreters and difficulty in booking interpreters via phone from some cultural backgrounds [15, 19]. In some cases, lack of appropriate access to interpreters resulted in CALD mothers using their friends, family members and bilingual staff to communicate with health service providers [18, 21, 22].

While a body of literature exists describing the issues experienced by clinicians, interpreters, and clients in primary health care nursing services, there is an absence of studies exploring strategies to address these and improve service quality. This study aimed to explore the experiences of healthcare interpreters working with CFHNs in providing CFHN services and SNHV programs to CALD families with limited English proficiency, in the context of a service development and improvement project.

## Methods

**Fig. 1:**
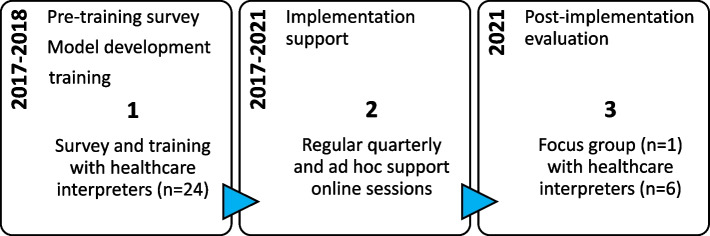
Study timeline

A mixed methods longitudinal research design was undertaken to design, implement and evaluate a training and practice support model for interpreters working with nurses and families in SNHV programs in three local health services in Sydney, Australia. The timeline for the study is presented in Fig. 1.

### Theoretical framework

This study is located within, and guided by, Bronfenbrenner’s [23] socioecological theory. The theory gives emphasis to the complex interplay between individual, interpersonal, organisational (e.g., community-level) and societal factors that shape service environments [24, 25]. The rationale for applying of the model is to address and evaluate the system and interpersonal factors influencing professional interpreter service delivery for CALD families with limited English proficiency. Socioecological theory has been used in previous research that has explored factors that facilitate or hinder access and engagement with health care services to migrant families [19, 26].

### Ethical issues

Ethics approval was granted from the Sydney Local Health District (SLHD) and South Eastern Sydney Local Health District (SESLHD) Ethics Committees (X17–0376 and HREC/17/RPAH/571; SSA 18/RPAH/116; SSA 18/G/078) and South Western Sydney Local Health District (SWSLHD) Ethics Committee (HREC/17/LPOOL/493).

### Data collection

1. Twenty-four interpreters who self-identified as frequently providing interpreter services for the SNHV service volunteered to undertake the training, completing a brief pre-training survey about their experiences and confidence in working in the home visiting context, including quantitative and open-ended responses about the interpreter’s role in home visits. All health services interpreters are certified, have completed an approved medical terminology course, abide by the NSW Health Code of Conduct, AUSIT Code of Ethics, and are recognised by the Commonwealth Government’s National Accreditation Authority for Translators and Interpreters (NAATI). A model of support was developed and training provided based on the findings of the survey, and field notes on the training were recorded by the implementation consultant/trainer. The implementation consultant/trainer was an experienced CFHN employed by the research group.
2. Throughout the implementation period (2017 to 2021) the implementation consultant/trainer maintained field notes based on regular (quarterly) and ad hoc requests for implementation support from either the interpreters and/or the nurses working with them.
3. One focus group was employed to elicit the experiences of interpreters for post-implementation evaluation. The participants were recruited by purposive pragmatic sampling via an email with a link to an online survey to complete demographic and practice information, should they wish to participate. Demographic data were obtained from a total of 7 interpreters. Of these, 6 interpreters participated in the focus group discussion, with one interpreter declining to take part due to work/personal issues. The 45 minute focus group discussion was carried out virtually (via Zoom platform) due to Covid-19 restrictions, moderated by the first author. The session was audio-recorded with permission from participants and transcribed verbatim.

### Data analysis

Quantitative survey data were analysed descriptively using Alchemer survey software. All qualitative data from across the study time-points were thematically analysed using the six phases of reflexive thematic analysis described by Braun et al. [27]: familiarization, generating codes, constructing themes, revising themes, defining themes, and producing the report. NVivo software supported the qualitative analysis. Interpretative rigour was enhanced by all authors checking themes against extracts of notes and focus group transcripts, making the research transparent and increasing credibility. The developed themes were reflected in the results upon mutual agreement.

## Results

### Pre-training survey and model development

**Fig. 2:**
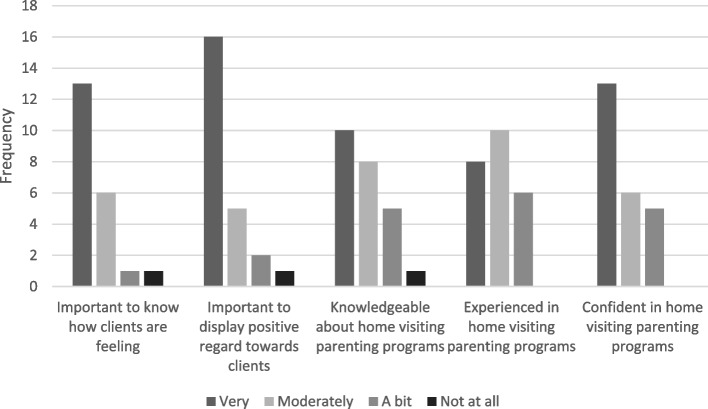
Interpreters’ survey reported attitudes, knowledge, experience and confidence

Most participant-interpreters had worked as an interpreter in the healthcare sector for more than 2 years (*n* = 22), and all had worked in community health. Twenty had home visiting experience. Three quarters of the interpreters felt knowledgeable about working in a program delivering an in-home parenting service to families, and nineteen interpreters felt confident about interpreter service delivery in that context. Most interpreters considered knowing how clients felt and displaying positive regard towards clients to be important. Figure 2 summarises the respondents’ reported attitudes, knowledge, experience and confidence. Trustworthiness and professionalism were considered by all interpreters to be important personal qualities for an interpreter involved in home visits.

Three themes were identified in the analysis of interpreter roles described in the open-ended responses to the pre-training survey (PTS). Most interpreters wrote about their role as being the provision of a translation service, expressed as:

*“To provide accurate interpretation of the conversation between the professional and the client.” (PTS)*

*“Facilitate communication and delivery accurately.” (PTS)*

*“Interpreting accurately to both parties.” (PTS)*

However, a smaller number of interpreters felt their role extended beyond supporting communication. Roles included were being a link, a cultural advisor or client advocate described as:

*“A bridge linking the clients and the healthcare practitioners.” (PTS)*

*“More as a facilitator than an interpreter, as well as a culture liaison person.” (PTS)*

*“The usual role of an interpreter - to interpret accurately and thoroughly, to provide cultural information when needed, to alert the health care provider [to] the subtle expression of patient's feelings.” (PTS)*

*“Interpreter, but at the same time an advocator of my (patient/client).” (PTS)*

Finally, some interpreters also described the importance of being able to *“make everybody feel comfortable” (PTS)* and create a feeling of mutuality when interpreting.

Based on these results, a model of training and support was developed that was designed to raise interpreters’ understanding of the SNHV program and to define the roles of the CFHN and interpreter in the home visiting context. Further, based on the identified desire for extended cultural roles by the interpreters, supporting comfort, and the importance to interpreters of knowing how clients are feeling and displaying positive regard, a model of pre-briefing and post-briefing between the interpreters and CFHNs was developed. The pre-briefing form was to be completed by the CHFN and sent to the interpreter by email, and included details such as the names, ages and roles of client family members (for example, children and grandparents) who were likely to be present at the home visit, and relevant details about the SNHV program content, family strengths and risks planned for discussion during the visit (for example, alerting the interpreter to potential discussions about family violence or child safety issues). The post-briefing form was to be completed by the interpreter and sent to the CFHN by email and focussed on the interpreter noting cultural issues that would be informative for the CFHN (for example, traditional dietary approaches to child weaning), as well as raising any concerns about the home visit, for example as one interpreter reported: *“Interpret and convey cultural conflicts and differences if present.” (PTS)*

### Training

The half day interactive training session included an overview of the SNHV program, structured interventions and program schedule, and the role of the interpreter when working with complex families in relation to the nurse as the primary worker with the family. Twenty-four interpreters in total participated in two training sessions in March 2017 and July 2018. During the training there were opportunities for the interpreters to question and raise points of concern that were noted in consultant/trainer field notes (FN).

Concerns about the need for nurses to have greater awareness of the role and limitations of interpreters were expressed with interpreters sharing,

*“So need to educate the nurses it’s not up to us what to say. We just say what is said and what the mother says and whatever is around it we cannot say anything other – I cannot add.” (FN)*

The interpreters also raised the issue that families/mothers may say *“Don’t tell the nurse” (FN),* with seven of the interpreters stating that they tell the mothers that they must tell the nurse, for example, by stating *“Whatever you tell me I have to tell the nurse” (FN)*. Interpreters also highlighted the need for both the interpreter and the nurse to understand practical issues like the additional time needed when delivering care with an interpreter, and the need to spend time building trust with clients using phrases, for instance *“Please trust us we are here to help you” (FN).*

The interpreters in the training appreciated the importance of continuity of carer that underpins the SNHV program, however, they had mixed experiences and concerns about interpreters providing continuity of service to clients. This was particularly of concern when the interpreters were from the same community and knew the families in other context, presenting privacy issues. They were also concerned about families becoming reliant on the one interpreter, and the risk to service engagement if the interpreter became unavailable.

### Implementation support

The issues raised in the survey and during the training suggested that, beyond training, a mechanism for ongoing support of the interpreters and CFHNs would be needed throughout the implementation of the practice development model, providing support for management of the roles and relationships. CFHNs and interpreters were offered regular quarterly and ad hoc support. These sessions focussed on particular issues that occurred during visits that caused stress to the interpreter and/or nurse. For example, one interpreter shared the challenge of working with multiple family members, as noted in a field note:

*“The interpreter gave an example of a mother and grandmother in an interchange - with the interpreter caught in the middle trying to relay what was happening to the nurse. The interpreter was stressed trying to interpret what was being said and capturing the continued conversation as she interpreted to the nurse. Also the interpreter was worried about upsetting the nurse because the grandmother didn’t like the information the nurse was sharing.” (FN)*

There were also issues related to the provision of interpreters with the correct language and/or dialect.

*“Also problems with assigning an interpreter but the language is not available and having to use e.g., Arabic when the language was Assyrian - I remember the Arabic interpreter sharing the problems of this. She spoke both languages and her care was in ensuring she used the correct language with the family which instantly gave her some rapport with Assyrian families.” (FN)*

Early implementation feedback reported that email, as the mechanism for pre- and post-briefing, was not functionally useful as the CFHN and/or interpreter could change on the day. Consequently, an online platform was developed for the nurses to provide the pre-briefing to the interpreter team, and for the interpreter to provide a post-briefing to the nurses. Subsequent notes from the implementation consultant/trainer revealed the platform was rarely used, with nurses and interpreters both raising issues of workload capacity; consistency of interpreter (for example, providing the briefing to one interpreter and a different interpreter attending the client’s home visit having not accessed the pre-briefing online); and nurse staffing changes making both nurses and interpreters feel that the effort of pre- and post-briefing was not worth the time invested. Subsequently, from early 2020, redeployment due to the COVID-19 pandemic exacerbated these issues such that pre- and post-briefings ceased entirely.

### Post-implementation evaluation

The interpreters who participated in the post-implementation evaluation were all female, aged between 38 to 69 years, and with professional work experience as an interpreter ranging between 10 and 43 years, with healthcare experience between 6 and 43 years. Current interpreted languages were participant reported as being Mandarin and Cantonese, Thai, Vietnamese, and Arabic. Four major themes were developed from post-implementation evaluation phase of the study including: system-related issues; interpreters’ challenges; working with nurses; and client session related issues.

#### System-related issues

The need for adequate time when interpreting a session; lack of same interpreter-client engagement in follow-up sessions; and issues with pre-briefing and post-briefing sessions were the most commonly identified system barriers for interpreters working with CFHNs in delivery of SNHV programs.

Some of the interpreters reported they managed program delivery without a pre-briefing session. Furthermore, their long-time experiences working in health care meant that they felt they knew most of the content and potential family issues, and they did not have any problems with the content of the delivered program. This was particularly the case where the interpreter had some continuity of engagement with families.

*“I have done enough of these appointments even like we don't have the pre-briefing for each appointment, but we have done like these appointments so many times over the years, so we're familiar enough [with] what to expect we just like, in general, what to expect in general just not particular clients.” (I5)*

*“In my case many of the families that we visited, I met them through antenatal clinic and because I am the only full-time one in my language group, so I met a lot of families, and I … it’s kind of … I would know them like the mother since the antenatal clinic, and then it’s kind of following up.” (I3)*

Nevertheless, interpreters highlighted the need for pre- and post-session briefing with nurses especially for mothers with mental health issues and/or greater family risk or complexity. Three interpreters indicated a lack of time for post-session debriefing, and they mostly needed to rush. Four interpreters reported there was no pre-briefing session or if there was, it was not adequate or provided early enough to cover the family’s information to facilitate their work.

*“I think for me most of the time, I had to rush to somewhere, so there was no debrief, [only] say hi, bye, thank you.” (I3)*

*“Mostly we don’t have much briefing, yeah, they just book, meet us outside the property and we just go in, with no pre-knowledge of the family”. (I2)*

*“I think it depends on the nurse. Sometimes yeah, going up to unit or before the appointment we have briefing like half a minute, or a minute yeah … , the nurse may give us very brief information about the parent and the child.” (I5)*

One of the interpreters strongly believed in the usefulness of having the same interpreter for one mother each time to better follow-up with mothers and their babies, but noted this was not happening as: *“now we are on the roster form, we just have to change interpreter every time, you just cannot follow up with a subsequent few visits.” (I1)*

#### Interpreters’ challenges

This theme focuses on the challenges experienced by the professional healthcare interpreters including: additional workload in interpreting mothers’ family members’; difficulty explaining interpreters’ perceptions due to mothers’ privacy issues; interpreters’ inconvenience matters; gender power; and privacy issues.

Some interpreters explained that their workload increases when they needed to interpret family members’ other than the principal client in the session.

*“Sometimes I find like if husband speaks to the nurse asks the questions in English they doubles up our work. It’s like I have to interpret what he said as well through the mum.” (I6)*

Some of the interpreters indicated that CALD mothers do not feel comfortable talking about private matters in front of male interpreters and the importance of working with female interpreters due to cultural and gender power issues.

*“Another thing in our culture, I don’t know in other culture … definitely it’s a female interpreter is the most important in those sessions.” (I4)*

*“Sometimes you turn up at family home and then the husband might say [to mother] why you have an interpreter, who is this person coming into my house?” (I3)*

#### Working with nurses

Most of the interpreters described some issues around working with CFHNs in provision of the services to CALD mothers. Some of the interpreters reported that CFHNs requested simultaneous interpreting. They believed that this mode of interpreting was not suitable for the CALD home visiting setting, especially for mothers with mental health issues, as mothers may get distracted and confused.

*“For my culture and I agree with you (I3) because simultaneously interpreting … it’s not for that setting and shouldn’t be, because it confuses most of my clients [mothers] and they don’t concentrate and you know, they’re looking at me and looking at nurse, very hard.” (I4)*

Two of the interpreters stated that they were sometimes asked by the nurse to share their cultural perceptions of what is being said during the session. In some circumstances it was difficult for them to explain their perception without breeching a mothers’ privacy, and some believed their role was only to interpret and help the communication.

*“What is missing is mostly like a cultural, like you don’t understand culture and then they may miss the cultural fit.” (I2)*

*“I am not there to explain what I have perceived because it’s very hard, we’re only interpreters, sometimes it’s very frustrating for us.” (I4)*

Some of the interpreters stated that sometimes they were asked by mothers to not relay some part of their conversation to the CFHNs. However, they stressed that they must translate the whole conversation to the CFHNs.

*“Many times, I just tell them that I am sorry whatever you said to me I have to translate to healthcare providers.” (I2)*

#### Client session related issues

The interpreters were reliant on the nurses to adequately manage the session time. One of the interpreters mentioned that although CFHNs were very good at controlling the time, scheduling delays occurred.

*“Normally we would be given an hour, yeah plus the travel time. But it [is] also depending on whether you have jobs before that and jobs after that, that you have to run too. Yeah, I mean most of the nurses control the time very well so within an hour all done. But I find it … sometimes because they’re doing several visits on the day, so when I reach my client house, they might be 10 minutes late.” (I3)*

The interpreters also emphasised the importance of CFHNs competency to organise and control the session well and although most of the interpreters reported that CFHNs mostly have good control of the session, some of them had negative experiences around lack of control of the session to support smooth interpretation.

*“Yeah, so it’s more about we can’t really do much. Cause we are just interpreters, but if the nurse could, you know … like organise well, and control that [the session] well then it would be helpful.” (I6)*

*“I worked with her [nurse] a lot, she [is] usually in very good control like, yeah, but somehow that day she didn’t take control, and she has a hearing aid so probably the noise didn’t bother her, and she is not the one straining the voice. She didn’t have this empathy, she didn’t realise [that] … oh I have been yelling for over [an] hour, yeah.” (I5)*

In addition, the interpreters described some matters of inconvenience such as concerns about the venues they attend or having difficulty interpreting in a noisy environment.

*“Dogs … when you get there. Some houses have dogs or actual addresses, sometimes it took a while before we could find it and [for example] then I had one visit actually it was on top of a brothel … ” (I3)*

*“In some cases, or many times, we had to … like interpret while the kids are around and making a lot of noise or crying. It’s a little bit difficult of course, but we try our best.” (I5)*

When asked about interpreters’ perceptions of an ideal session, all interpreters described the following requirements: to have a safe and private environment for interpreting the session; on-time sessions; having a feeling of accomplishment by delivering quality services (to mothers and nurses); and informing mothers of available services including receiving extra services such as nurse home visiting.

*“I think the ideal is, such as … like the client gets what they want, and the nurses get what they want and I've been helping them to get what they want, then I feel just accomplished.” (I2)*

## Discussion

This study demonstrates that interpreters’ experiences of working with CFHNs in providing SNHV programs to CALD families are challenging. Pre- and post-briefing and adequate time were identified throughout all phases of this service development project as key to improving interpreting quality, particularly the capacity to provide cultural bridging and adequately support client, interpreter and CFHN comfort. Pre- and post-briefing were identified as key strategies to support quality mental health services in a recent narrative review [28]. However, system issues around time, workforce deployment and capacity remain major barriers. This is consistent with findings of previous research that quality of interpreting is negatively impacted by lack of time, and system barriers to access to interpreters [10, 29]. Some interpreters’ long-time experiences and pre-knowledge about some CALD mothers from antenatal clinics enabled them to manage the interpreting sessions without receiving pre-briefing sessions. This would not, however, be the case for CALD mothers new to the service, and especially those in more complex situations, such as mothers with mental health issues. This study suggests allocating sufficient time for interpreting during client home visits, and pre- and post-briefing are critical to improve the nurse-mother-interpreter interaction and subsequently to improve quality of care.

In addition, the issue was raised by some interpreters that CFHNs have expectations of cultural input from interpreters, and indeed, some interpreters themselves indicated that they felt that they had a broader role of providing comfort and cultural knowledge and connection, within the context of a session. However, their role cannot and should not be extended beyond communication to being a bridge linking the families and CFHNs. This study suggests that interpreters’ uncertainties to disclose their perceptions with attention to CALD mothers’ cultural beliefs and customs of the family needs to be addressed, particularly as the CFHNs are not usually of the client’s culture. It is consistent with research findings that interpreters’ uncertainty and lack of clarity about their roles and responsibilities beyond literal interpretation [10, 30, 31] may lead to confusion and lack of trust between clinicians, clients and interpreters [10]. Trustworthiness was identified by the interpreters in this study as an important personal quality for interpreters, and trust is a core facet in long-term relationship-based primary care nursing, typified in SNHV programs.

Although interpreters described some positive experiences with CFHNs through this longitudinal study, some factors complicated their work including privacy and gender issues, considering CALD mothers’ culture in choosing the interpreting mode, and lack of cultural compatibilities between the mother and nurse. Interpreters strongly agreed on the importance of working with female interpreters. This finding is consistent with previous research that revealed working with female interpreters positively influenced migrant women’s access to maternity care and was critical in supporting them to disclose women’s health or family concerns [18].

Continuity of care and relationship between the family and CFHN is a key component of SNHV, but interpreters were divided about whether continuity of interpreter was beneficial. There were system issues that mitigated against continuity when it was desired, and privacy concerns within smaller CALD communities, as well as stress associated with potentially being “caught in the middle” of family and family-CFHN relationships. These findings were in contrast to previous research that endorsed interpreter continuity [26, 28, 29]. The findings of this study suggest that it would be inappropriate to recommend a single practice with regards to interpreter continuity, but rather, that the nuances of community size and interpreter-CFHN-family relationships need to be considered.

Such challenges were expected to be addressed through the training, pre- and post-briefings, greater planning, and appropriate continuity of interpreter, which were desired by the interpreters, but which were difficult to implement in these services at this time.

### Strengths and limitation

This study described healthcare interpreters’ experiences and perceptions in working with nurses in both CFHN services and SNHV programs with a specific population of CALD mothers in the context of a longitudinal practice improvement program. In addition, collecting data from specific time periods allows noting of similarities and differences across time and implementation. This research represented a small number of female interpreters from three sites, with no male interpreters participating in this study. Nevertheless, this study’s findings are consistent with the limited international research.

## Conclusion

The findings of this study highlight that interpreters’ perceptions of challenges and their unmet needs and supports for working with CFHNs, in delivery of sustained and relationship-based services to CALD families with young children, have been consistent over time and remain resistant to attempts at service improvement. To achieve better access and engagement of CALD mothers with services, it is crucial to make system changes to allocate sufficient time, better coordination of interpreting sessions (including pre-briefing and post-briefing sessions), clear statements of interpreter roles, that respect their desire to be more than literal translators and provisioning for interpreters’ safety during home visits with nurses. Furthermore, the appropriate use of interpreters, specifically female interpreters, and the same interpreter for continuity with each CALD family in the SNHV program requires further consideration. System research is needed to explore the barriers and potential facilitators of much-needed system changes to improve interpreters’ experiences and service quality for families receiving SNHV program and other relationship-based community and primary health care services.

## Data Availability

The data that support the findings of this study are available on request from the corresponding author. The data are not publicly available due to privacy or ethical restrictions.

## Abbreviations

CALD: Culturally and linguistically diverse
CFHN: Child and family health nursing
CFHNs: Child and family health nurses
FN: Field notes
I: Interpreter
LHD: Local Health District
PTS: Pre-training survey
SNHV: Sustained nurse home visiting
